# Innovation at the Intersection of Alcohol and HIV Research

**DOI:** 10.1007/s10461-017-1926-z

**Published:** 2017-10-17

**Authors:** Alastair van Heerden, Mark Tomlinson, Sarah Skeen, Charles Parry, Kendal Bryant, Mary Jane Rotheram-Borus

**Affiliations:** 1 Human Sciences Research Council, 22 Mbuvu Dr, Sweetwater, 3201 Pietermaritzburg, South Africa; 2 MRC/Wits Developmental Pathways for Health Research Unit, University of the Witwatersrand, Johannesburg, South Africa; 3 Department of Psychology, Stellenbosch University, Stellenbosch, South Africa; 4 Alcohol, Tobacco and Other Drug Research Unit, Medical Research Council, Cape Town, South Africa; 5 Department of Psychiatry, Stellenbosch University, Tygerberg, South Africa; 6 National Institute on Alcohol Abuse and Alcoholism, Rockville, MD USA; 7 Department of Psychiatry and Biobehavioral Sciences, Semel Institute for Neuroscience and Human Behavior, University of California at Los Angeles, Los Angeles, CA USA

**Keywords:** Interdisciplinary research, Alcohol abuse, HIV and AIDS, Technology, Conversational agents, Virtual and augmented reality, Funding models

## Abstract

Working in an interdisciplinary manner at the crossroads of alcohol and HIV research is a challenge. This paper presents six novel approaches that could be applied to activities at the intersection of alcohol and HIV. These approaches are (i) address the fact that the availability of new technology is unevenly distributed around the world, (ii) use technology to move beyond both paper and digital surveys, (iii) introduce a focus on advocacy and partnerships with large technology companies, (iv) harness technological innovation to utilise digital counselling, (v) explore the use of virtual reality in both research and delivering interventions, and (vi) consider alternative funding models to those currently in existence to improve efficiencies and innovations. Aiming to understand the interplay of alcohol and HIV will require creativity. The six approaches outlines in this paper provide possible directions from which new approaches may emerge.

## Introduction

The use of alcohol impacts on many people, including families and those infected by and at risk of acquiring HIV. Alcohol use, the drinking of alcohol, is a critical behavioural risk factor, and both the volume of alcohol consumed and the pattern of drinking can lead to the reduced efficacy of both behavioural prevention and biomedical prevention and treatment strategies [1]. Alcohol use has repeatedly been shown to elevate sexual risk associated with sexually transmitted infections [2]. Mechanisms include impaired judgement, delayed testing and challenges following complex medical regimens. Alcohol use can also accelerate HIV disease progression as well as increase HIV transmission through risky sexual behaviour, HIV shedding and mucosal inflammation [3].

While these data suggest a clear advantage is to be gained from considering alcohol and HIV concurrently, working at the intersection of these two fields, both with well-established literature and methodologies, is a challenge because of issues such as the use of different lexicons and the lack of high quality interdisciplinary journals to publish in [4]. There is merit to drawing on the best of current approaches in each field as well as taking the opportunity to explore novel and innovative new strategies to gather information, to fund research, and to deliver interventions. This paper presents six approaches that could be applied to activities at the intersection of alcohol and HIV. These approaches are (i) transfer and translate new and unevenly distributed technologies, (ii) use technology to move beyond both paper and digital surveys, (iii) introduce a focus on advocacy and partnerships with large technology companies, (iv) harness technological innovation to utilise digital counselling, (v) explore the use of virtual reality in both research and delivering interventions, and (vi) consider alternative funding models to those currently in existence to improve efficiencies and innovations. While many of the approaches described are applicable beyond the intersection of alcohol and HIV, discussing them within this framework allows for concrete examples of each approach to be made.

## Transfer and Translate New and Unevenly Distributed Technologies

At times innovation is incremental and at other times change is punctuated by rapid and abrupt disruption that significantly alters the landscape [5]. In both instances, the diffusion of innovation is often asymmetrical. For example, while the internet became available in high income countries in the 1990s, internet speeds and broadband access continues to lag in Africa [6]. Many promising substance abuse interventions that work well in high resource settings are impractical to positively impact on the lives of those most affected by HIV and AIDS. For example, Anand et al. [7] report on an eHealth prevention service that leverages an online HIV education and counselling website for men who have sex with men and transgendered women communities. Site users who are interested in pre-exposure prophylaxis (PrEP) are linked with facilities where they can take an HIV test and collect PrEP. The authors find this model to be highly effective, with a threefold increase in PrEP uptake among participants who first access HIV educational and counselling information through the website.

Translating and tailoring these online intervention programmes for use in low and middle income countries (LMICs) would require significant adaption as both services providers and users are generally not online themselves, and although mobile phone penetration in Africa, for example, is over 80%, internet access is still limited and unstable (estimated at 29.2%) [8]. This introduces a different set of design challenges and considerations that must be addressed before these online platforms can be effectively harnessed for people at risk and living with HIV in LMICs. One limitation of this approach is that certain online tools may only work as intended in a high bandwidth environment. Adaption for offline or low-connectivity use may be impractical.

## Moving Beyond Surveys to Direct Measurement

Collecting accurate self-report survey data on either alcohol use and/or sexual practices can be a challenge [9]. People tend to present themselves in the most favourable light which can introduce bias when asking about sensitive issues. Self-reporting of sensitive (e.g., drinking by pregnant women or persons taking medications) and/or illegal (e.g., heroin use) activities has produced mixed findings with some studies finding limited effects [10], with other suggesting that impression management can reduce reporting by as much as 50% [11]. Research legitimacy, cognitive burden and perceptions of privacy all interact with survey administration mode (self vs interviewer-led) to impact participants’ wiliness to accurately respond to questionnaire items [12]. Direct measurement using breath alcohol level or blood alcohol concentration is also limited by rapid metabolisation of alcohol. An alternative non-invasive, passive and accurate approach to assess behaviour such as alcohol or drug use involves the use of transdermal biomarker sensors. While the technology has been available for some time [13], next generation devices use microneedle arrays to monitor molecular markers in the interstitial fluid. These devices make the real-time, continuous monitoring of inferred blood alcohol concentrations a possibility [14, 15]. This is achieved by engineering a surface that feels smooth but is in fact covered by thousands of micron-sized needles made of silicon, glass, metal or some form of biodegradable polymer. The application of this surface to the skin allows macromolecules to pass the skin barrier between device and interstitial fluids without any experience of pain [16].

Recalling the number of times one has had sex in the last 3 months can be equally unreliable. While ethical issues would first need to be addressed, technology such as the Electronically Activated Recorder (EAR) and suitably trained models could turn audio snippets into a count of sexual episodes before discarding the audio [17, 18]. The EAR is an app installed on a smartphone that automatically records brief audio samples repeatedly through the day (usually around every 12 min). No participant action is required as the EAR is a passive approach to data collection. An alternative, complimentary approach, may be recoding physiological changes that take place during sexual intercourse such as flushing. While these approaches may offer novel and interesting possibilities for data collection, the costs may outweigh the benefits in certain circumstances.

## Advocacy and Partnerships between Academia and Corporations

Large technology companies are hiring research scientists out of academia at an increasing rate [19]. This shift has been driven by the availability of large user generated data sets and the realisation that these data can be used to drive profit. Rather than large, multi-year, one off Randomised Control Trials (RCTs) companies such as Facebook, Amazon and Uber conduct thousands of small incremental experiments on their networks daily [20]. While a multi-country intervention trial with 1 billion participants is beyond the scope of any academic research study, it is increasingly feasible for many large technology companies. Global health research undertaken for public good should not ignore this, but rather advocate for partnerships to be formed between the owners of these socio-behavioural datasets and academic research undertaken using traditional study designs. This could lead to a mutually beneficial relationship with expertise in study design, ethics and good clinical practice being valuable contributions academic research could offer [21]. Managing the potential conflicts of interest between the researchers and the company objectives of maximizing profits is one limitation to this approach.

## Harness Technological Innovation in Approaches to Counseling

Digital assistants such as Apples’ Siri are currently limited in their abilities. However, they suggest potential future applications of digital assistants to both HIV and substance use counselling [22]. Adaptive interventions could be designed that first offers information and basic support through a virtual conversational agent (sometimes called virtual avatars or “chatbots”). These agents are able to engage in back and forth dialog with users in conversations that feel as if one is talking to a real person). The virtual agent could escalate the session to a counsellor only when necessary thereby helping to scale the benefits of counselling not possible using the current one-on-one individual counselling model.

**Fig. 1:**
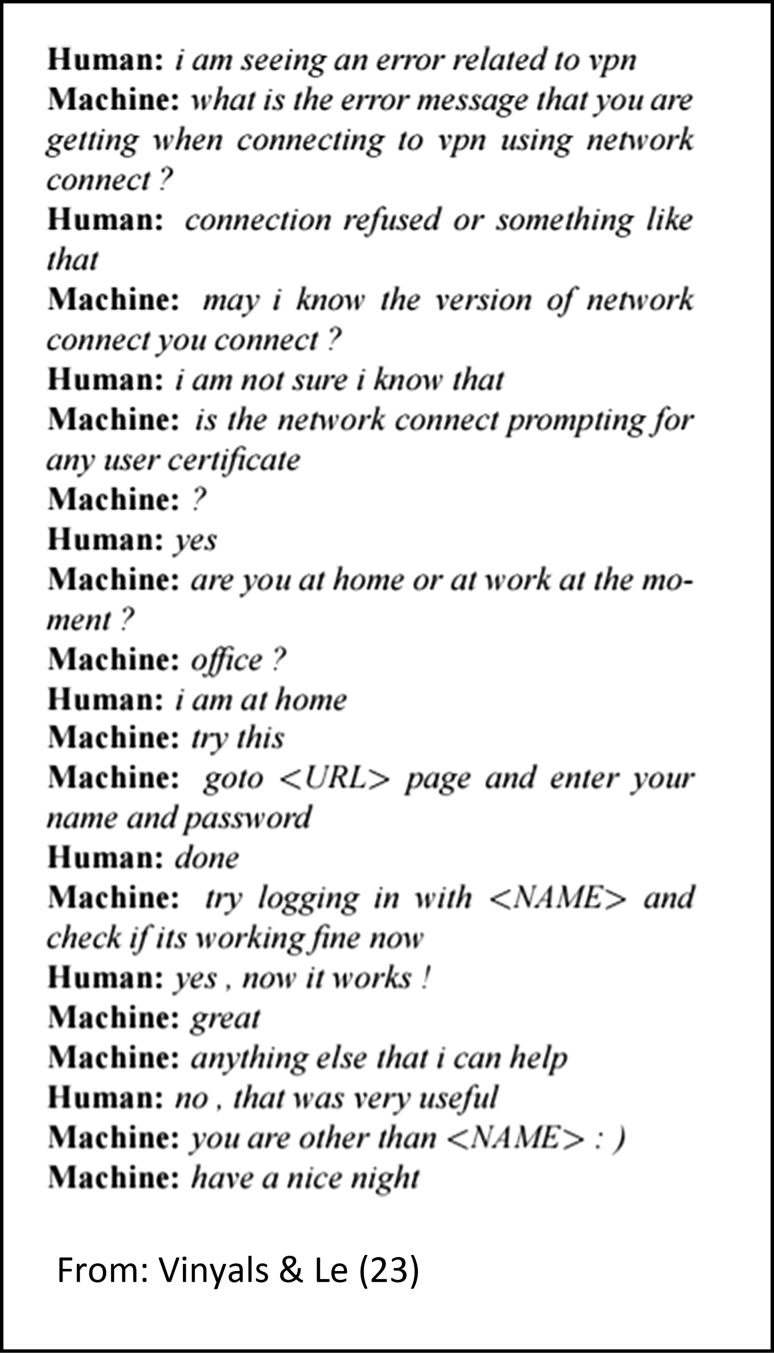
Example conversation between human and computer generated conversation *From* Vinyals & Le [23]

While counseling seems a very human skill, recent evidence suggests that with a large enough set of data, it may be possible to model realistic conversations without needing to rely on the old approach of predefined IF–THEN rules [23]. For example, IF [the user says I am HIV positive], THEN say “I am sorry to hear this, when did you find out”. The weakness of this approach is that it is very fragile and being prepared for all possible user responses is not often feasible. Instead, the conversation in Fig. 1, shows another approach [23]. All agent responses in the back-and-forth dialog were generated without any hand crafted rules. Instead, the agent reviewed a large number of previous help desk conversations and learnt from these data what might be an appropriate response to different user requests. It then generated its responses to the questions posed by the user on–the-fly. In other work, using an online counseling dataset consisting of 3.2 million messages sent by 408 counselors, [24] were able to construct a model able to predict, with an accuracy of 59%, the likelihood of a counseling session ending positively, after reviewing the first 20% of a conversation between a client and counselor. As more data become available, conversational agents will continue to improve and become realistic additions to intervention research [22]. Until the technology matures, upfront costs in terms of time, money, and risk may limit the uptake of conversational agents in research.

## Conducting Research in Virtual Reality Environments

The possibility of either augmenting reality or immersing participants in virtual reality could help address some of the challenges currently faced in alcohol research [25]. Augmented reality is the use of a screen, such as a pair of glasses, goggles or mobile phone screen held as if taking a picture, to generate and place 3D digital objects in the real world. On the other hand, virtual reality requires a headset that completely obscures the real world and immerses the wearer in a virtual world [26]. Both approaches may have application to HIV and alcohol research. For example, current methods of understanding drinking behaviour among persons at risk of acquiring HIV or who are already affected and possibly on antiretrovirals (ARVs) is often limited to survey research [27]. The challenge of this approach is given that people, in a single drinking occasion, can drink different kinds of beverages using different sized containers and even drink communally, how does one accurately measure consumption? Using augmented or virtual reality, a new methodology may be available for understanding these situations and assisting a person in identifying what they have consumed and in what quantities, and could thus be an aid to screening, brief interventions and referral to treatment in primary health care, HIV clinics and other settings [28]. Another possible use of this technology may be in resistance skills training in preventing/reducing substance [29]. Virtual reality simulations could be used to present various scenarios to the child and/or adolescent in which they are exposed to temptations to drink and they could be coached on how to respond in each situation. They could also be evaluated on their ability to resist pressures by being presented with further scenarios once training has been completed. This approach could also be used to guide adults on how to escape social pressure to drink more in situations that put them at risk for acquiring HIV or that challenge their ability to adhere to their antiretroviral medications. Scare availability of the skills required to build virtual environments may limit the use of virtual reality in research.

## Consider Alternative Funding Models for Research

Just as researchers explore alternative interdisciplinary approaches to their work, with the intent of fostering innovation and creativity, funders too might consider how deploying alternative funding strategies may impact the type of research that can be undertaken in the alcohol and HIV areas. While incremental change is the predominant approach to innovation, disruptive step-level change does and can occur. These ideas are often currently lost due to a system that is designed for the incremental approach. New ideas, if not backed by existing data are harder to get funded. Current funding practices encourage scientists to take nearly completed projects, re-package them with a new coloured bow and a slight twist and submit them for review [30]. The process encourages safety and rewards those who already have funded work. Further, huge amounts of time are used up preparing, submitting and reviewing one’s own and others’ proposals. Bold funding initiatives that back wild ideas may be necessary. Bollen et al. suggest one such alternative approach [31]. In summary, each scientists meeting certain entry criteria are funded by national funding agencies to a predefined value (for example $250,000). The scientist receiving the award must pass a portion of the money, for example, 50%, on to one or more colleagues in their field. Each scientist would set their own criteria such as originality, quality and relevance, for evaluating whom to pass the money on to. The scientist receiving the funds would similarly need to pass on a portion of the funds to a scientist of their choice. This model was shown by Bollen et al. to result in a funding distribution favoured by the entire scientific community, all without a single grant proposal needing to be written or reviewed. A final advantage of such a system is that scientists with strong ideas, no matter how innovative, can be funded rather than the current “project model” which introduce the already mentioned limitations and biases. This and other non-traditional approaches to funding (such as crowdfunding) emphasises flexibility and openness, aligning more closely with agile, multidisciplinary and emergent nature of some contemporary research initiatives. Such funding systems could promote collaboration on new HIV and alcohol initiatives that require alternative funding models.

## Conclusion

Some of the ideas presented above are unconventional and challenge well-established ways of doing things. Bringing two fields as large as HIV and alcohol together can only be achieved by creatively exploring some of these new ways of working. By stepping back from business as usual it may become possible to see opportunities that were not obvious from within only the confines of one field and one approach and in terms of accessing data and undertaking interventions. If we are to be successful, ‘hairy problems’ like reducing alcohol use and improving HIV prevention will require creative structural solutions, such as those laid out in this paper.
